# Clinical, Epidemiological, and Histopathological Features of Respiratory Involvement in Rheumatoid Arthritis

**DOI:** 10.1155/2017/7915340

**Published:** 2017-11-07

**Authors:** Alessia Alunno, Roberto Gerli, Roberto Giacomelli, Francesco Carubbi

**Affiliations:** ^1^Rheumatology Unit, Department of Medicine, University of Perugia, Perugia, Italy; ^2^Rheumatology Unit, Department of Biotechnological and Applied Clinical Science, School of Medicine, University of L'Aquila, L'Aquila, Italy; ^3^Department of Medicine, ASL 1 Avezzano Sulmona L'Aquila, L'Aquila, Italy

## Abstract

Although by definition rheumatoid arthritis (RA) is an articular disorder, it is a systemic disease, and 18–40% of patients experience extra-articular manifestations (EAMs). The involvement of the respiratory system occurs in about 30–40% of RA patients, and in about 10–20% of them it represents the first manifestation of RA. A wide range of pulmonary manifestations are detectable in RA patients, including pulmonary parenchymal disease, pleural involvement, and airway and pulmonary inflammation. The clinical, radiological, and histological spectra of respiratory manifestations in RA reflect chronic immune activation, increased susceptibility to infection (often related to immunosuppressive medications), or direct drug. The type and severity of pulmonary involvement influence the prognosis, ranging from mild self-limiting conditions to severe life-threatening complications. Herein, we reviewed the various manifestations of respiratory involvement in RA, providing an overview on epidemiological, histological, clinical, and radiological data.

## 1. Introduction

**Figure 1:**
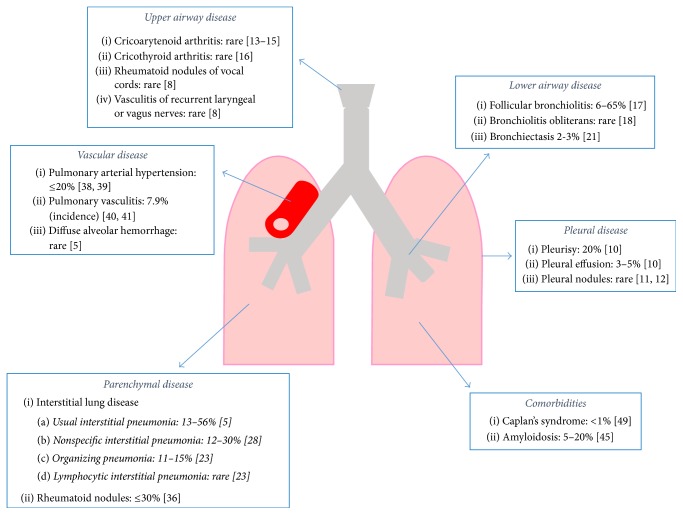
Respiratory manifestations in rheumatoid arthritis. Percentages indicate disease prevalence unless otherwise stated. Numbers in round brackets indicate the corresponding reference.

Rheumatoid arthritis (RA) is a chronic inflammatory disease of the musculoskeletal system with a clinical picture dominated by signs and symptoms of joint involvement [1]. The hallmark of the disease is symmetric chronic synovitis mainly affecting small peripheral joints, but virtually all joints equipped with a synovial membrane can be involved. Although this inflammatory process is reversible, it may evolve into irreversible damage of articular structures if left untreated and may lead to loss of function [2]. Although RA is by definition an articular disorder, it is a systemic disease. In fact, in a consistent subgroup of patients, extra-articular manifestations (EAMs) may occur, affecting other organs and tissues and thereby worsening disease prognosis. The incidence of EAMs is widely variable across different studies. Overall, EAMs occur in 18–40% of RA patients, while the incidence of severe EAMs ranges from 1 to 20%. Interestingly, EAMs may often be the first presentation of the disease before any articular involvement. Furthermore, extra-articular sites can also be the target of comorbidities (e.g., cardiovascular (CV) manifestations) or of the damage induced by RA-related drugs (e.g., corticosteroid-induced osteoporosis), requiring therefore an accurate differential diagnosis [3]. The involvement of the respiratory system occurs in about 30–40% of RA patients, and it is the first manifestation of the disease in about 10–20% of them (Figure 1). Pulmonary manifestations in RA mainly reflect the involvement of interstitium, airways, and pleurae, while vascular involvement is less frequent and diffuse alveolar hemorrhage (DAH), when observed in these patients, has a different etiology [4]. Overall, pulmonary manifestations account for 10–20% of mortalities in RA [5] and have been recognized as the second most frequent cause of death in RA patients, after CV disease [6].

On this basis, it is important to understand the pathogenic mechanisms underlying any respiratory manifestations in RA patients, in order to recognize their relationship with the disease or the treatment and to possibly design specific and effective therapeutic approaches. The aims of this review article are to provide an overview of epidemiological data on respiratory involvement in RA and to highlight the progress achieved so far in the understanding of pathogenic mechanisms and in the identification of therapeutic applications and implications needing to be addressed in future studies.

## 2. RA-Induced Pulmonary Involvement

### 2.1. Pleural Disease

Pleural involvement is common in RA with a prevalence of up to 50–70% in autoptic studies [7]. Symptomatic pleural involvement is observed in 3 to 5% of RA patients but signs of previous pleurisy are often detected in RA patients without a history of respiratory symptoms [8]. Pleurisy, pleural effusion, and pleural nodules are the most common manifestations, while pneumothorax is rare [9]; fibrothorax has been reported as a complication of recurrent untreated and severe pleurisy. It is not unusual that pleural effusion and pericardial effusion occur together [10]. These manifestations are more often observed in adult male RA patients with a high titer of rheumatoid factor (RF) [10]. Moreover, several studies demonstrated that pleural effusion and pleurisy typically occur as a late RA manifestation often associated with subcutaneous rheumatoid nodules [11]. The majority of fluids accumulating in the pleural space are derived from the lung through the visceral pleura and absorbed primarily through the parietal pleura. In inflamed pleura, this mechanism of fluid resorption is hampered, and in addition, the release of inflammatory mediators leads to endothelial injury and increased capillary permeability [8]. Therefore, RA-associated pleural effusion is typically an exudate and in more than 70% it is unilateral (with left predominance). Pleural thickening is often observed in biopsy specimens, due to the replacement of normal mesothelial cells with a layer of epithelioid cells and intercalated focal multinucleated giant cells. Structures resembling rheumatoid nodules can also be observed, while typical rheumatoid nodules are rare to find [11, 12]. In most cases, RA patients with pleural effusion remain asymptomatic due to the small amount of fluid that can only be detected on chest radiography as blunting of costophrenic angles [10]. Well localized chest pain aggravated by cough and respiration as well as dyspnea may appear if the fluid volume is big enough to stimulate parietal pleura sensory nervous fibers.

### 2.2. Airway Disease

Both upper and lower airways can be involved in patients with RA [8]. With regard to upper airways, laryngeal involvement has been described. Arthritis of the cricoarytenoid joint is rare but it deserves attention as the clinical spectrum ranges from mild dysphagia, throat pain, or dyspnea upon exertion to sudden glottic stenosis, stridor, and acute respiratory failure requiring immediate surgical treatment [13–15]. Similar to the involvement of any other joint equipped with a synovial membrane, cricoarytenoid joint arthritis is characterized by synovial thickening and joint effusion and eventually leads to cartilage erosion and joint subluxation as demonstrated by high-resolution computed tomography (HRCT) [13]. It is interesting to note that also the cricothyroid joint can be affected by RA and lead to vocal cord fatigue without affecting airway function [16]. Other RA manifestations involving upper airways are rheumatoid nodules of the vocal cords and vasculitis of the recurrent laryngeal or vagus nerves leading to vocal cord paralysis [8]. With regard to lower airways, the prevalence of bronchiolar involvement ranges from less than 10% to over 60% [17] and it can present as follicular bronchiolitis or constrictive bronchiolitis obliterans. In both cases, narrowing of the lumen is observed, but in follicular bronchiolitis it results from bronchial associated lymphoid tissue (BALT) hyperplasia, while in constrictive bronchiolitis obliterans it results from the development of concentric fibrosis [5, 17]. Follicular bronchiolitis is a milder disease dominated by an inflammatory pattern, with germinal center-like structures being the peculiar histopathological feature. Pulmonary function tests (PFTs) show a mild restrictive disease not requiring any specific treatment besides that of RA, and HRCT reveals wall thickening and centrilobular peribronchial nodules. Conversely, constrictive bronchiolitis obliterans is a severe condition presenting with rapidly progressing dyspnea and bronchorrhea. It often occurs in female patients with high titers of RF with untreated long-standing RA [18]. PFTs show an obstructive pattern and HRCT reveals wall thickening, centrilobular emphysema, and bronchiectasis. Despite being fatal in the majority of cases due to respiratory failure within months to years, some data reported efficacy of macrolide antibiotics [19] and tumor necrosis factor-*α* (TNF-*α*) blockers [20].

The estimated prevalence of bronchiectasis in RA is 2-3%; however, such abnormality can be detected by HRCT in up to 30% of asymptomatic patients with normal chest X-ray. Furthermore, up to 75% of RA patients with chronic respiratory symptoms or abnormal chest radiographic findings display traction bronchiectasis at HRCT [21]. Smoking and severe recurrent infections of the lower airways are well established risk factors for bronchiectasis. However, the actual etiopathogenic mechanism, including the possible role of RA specific drugs, is still a matter of debate [22]. Nonetheless, conventional synthetic (cs-) and biologic (b-) disease modifying antirheumatic drugs (DMARDs) should be used with caution in patients with bronchiectasis as both of them predispose to infections [8]. Furthermore, RA patients with bronchiectasis have higher mortality rates compared to RA patients without this manifestation and to patients with bronchiectasis without RA [22].

### 2.3. Parenchymal Disease

#### 2.3.1. Interstitial Lung Disease (ILD)

Being a frequent cause of death in patients with RA, ILD is the pulmonary manifestation requiring most attention [6]. The reported prevalence ranges from 4 to 70% according to different cohorts [23] and the clinical, radiological, and histological spectra of ILD are wide, including conditions characterized by an inflammatory infiltrate susceptible to corticosteroid/immune suppressant treatment to rapidly progressing fibrotic conditions with poor response to therapy and low survival rates. ILD usually occurs as a complication in long-standing disease; however, it may also be the presenting manifestation in up to 10% of patients [24]. Genetic predisposition, namely, the shared epitope [25], and a higher prevalence in smoker, seropositive, male patients have been also demonstrated [26, 27]. As far as etiopathogenesis is concerned, some studies put forward the hypothesis that inflammation may be an early event in ILD with the recruitment of immune cells to the lung interstitium, followed by epithelial and endothelial damage. In this phase, the process is still reversible and therefore prompt diagnosis and treatment lead to the resolution of inflammation with few or no sequelae. However, if the process moves forward, the subsequent event is an aberrant attempt to repair inflammation-induced damage with the activation of resident fibroblasts/myofibroblasts and the so-called “epithelial-mesenchymal transition.” Fibrotic response is then triggered, leading to a progressive and irreversible derangement of micro- and macroarchitecture of the lung and impairment of lung physiology until respiratory failure within months/years [28]. Smoking is a well-established environmental trigger stimulus for lung inflammation and it has been associated with ILD [29]. In RA, the combination of local and systemic inflammation together with persistent underlying immune cell activation cooperates to induce the development of ILD. As far as the autoimmune response is concerned, it has been known since the 1970s that RF is able to worsen pulmonary inflammation in experimental models [30], and more recently also anticitrullinated peptide antibodies (ACPA) have been associated with ILD [26]. Although smoking and ACPA are linked, as the enzyme responsible for protein citrullination is induced by smoking, the observation of ACPA in the bronchoalveolar lavage (BAL) of nonsmoking RA patients clearly opens several scenarios about other factors inducing the development of ACPA and therefore of ILD [31, 32]. Among all the ILD patterns, usual interstitial pneumonia (UIP) and nonspecific interstitial pneumonia (NSIP) are the most frequent, accounting for 40–60% and 11–30% of cases, respectively [28]. Biopsy specimens show that NSIP is characterized by diffuse alveolar septal thickening with lymphoplasmacytic inflammation and accentuation around bronchioles. Varying amounts of interstitial inflammation and fibrosis with a uniform appearance can be detected, and in most cases NSIP has a predominant fibrotic pattern rather than an inflammatory pattern [33, 34]. Alveolar spaces and subpleural areas are free of inflammation. In UIP, lesions are fibrotic (microscopic honeycombing), typically patchy with the subpleural areas more affected than central areas, and lesions are at different stages of development. Fibroblast foci, revealing areas where the fibrosis process is starting, can be detected at the interface between peripheral and central areas [35]. With regard to HRCT, NSIP is characterized by bilateral, symmetric, basilar, peripheral ground glass opacities, intra- and interlobular septal thickening, and reticular opacities as well traction bronchiectasis. Subpleural areas are typically free of lesions. Conversely, UIP appears with bilateral, basilar, subpleural fibrosis with architecture derangement, subpleural cysts, and traction bronchiectasis [23]. Dyspnea upon exertion is the peculiar clinical feature of RA patients with ILD, but it might be underestimated in patients with comorbidities such as chronic obstructive pulmonary disease (COPD) or, on the other hand, may subtend another pulmonary manifestation.

Of note, the prognosis of ILD associated with RA or other connective tissue diseases is better than that of idiopathic ILD [36]. In this regard, it was believed that the radiographic pattern, in terms of features and extension, was the main determinant of survival in RA-ILD; however, recent data suggest that greater PFT impairment and evidence of rapid disease progression over time rather than the chest imaging pattern are the most reliable predictors of survival in RA-ILD patients [37].

Although other ILD patterns are less frequent in RA, still they deserve to be mentioned in this context. Organizing pneumonia (OP) is characterized by polyps/granulation tissue of loose organizing connective tissue protruding into the bronchiole lumen and surrounding alveolar ducts and spaces. Thickening of alveolar septa and chronic inflammatory infiltrates can also be observed, and lesions are typically patchy and surrounded by areas of normal parenchyma [5]. At HRCT imaging, a dynamic pattern changing over time is peculiar of OP with patchy/nodular subpleural/peribronchovascular airspace consolidation surrounding areas of ground glass opacities [23]. Interestingly, an OP pattern can be superimposed to another ILD pattern (NSIP or UIP), and when observed alone it may occur prior to RA diagnosis [38]. Lymphocytic interstitial pneumonia (LIP) owes its name to the prominent mononuclear cell infiltrate observed in biopsy specimens [5]. Such infiltrate may organize in GC-like structures and lead to the thickening of alveolar septa. LIP is often associated with follicular bronchiolitis, and although it is detected also in RA, it is encountered far more frequently in primary Sjögren's syndrome [39]. HRCT findings are ground glass or centrilobular nodules, septal/bronchovascular thickening, and perivascular thin-walled cysts [23].

#### 2.3.2. Rheumatoid Nodules

Rheumatoid nodules are a peculiar feature of seropositive long-standing RA and, besides the subcutaneous localization, they can often develop in the lungs [40]. The size is highly variable from few millimeters to several centimeters, and they remain asymptomatic unless complicated (e.g., cavitation and superimposed infection). Rheumatoid nodules can coexist in up to 30% of patients with ILD [5]. Histopathological features of rheumatoid nodules include a central area of brightly eosinophilic necrosis surrounded by a zone of dark basophilic necrosis and more peripherally by palisading histiocytes. Signs of vasculitis can also be detected. Given some shared features with necrotizing granulomas, a careful differential diagnosis with infectious and granulomatous diseases is advisable [5].

### 2.4. Vascular Disease

#### 2.4.1. Pulmonary Arterial Hypertension

Pulmonary arterial hypertension (PAH) is a clinical condition characterized by the presence of precapillary pulmonary hypertension (PH) in the absence of other causes of precapillary PH such as PH due to lung diseases, chronic thromboembolic PH, or other rare diseases [41]. The exact prevalence of PAH in RA is not known, but it is rarely observed, mostly in older patients with long-standing disease. Interestingly, studies reporting higher prevalence of PAH in RA (up to 20%) through echocardiography evaluation have been performed in asymptomatic patients, which raises the question on how clinically and prognostically relevant the observation of increased pulmonary pressure is in these patients. The diagnosis of PH due to lung disease is instead more frequent in RA and, unfortunately, effective therapeutic approaches for this condition are lacking [42, 43].

#### 2.4.2. Pulmonary Vasculitis

Systemic vasculitis is a well-known but rare EAM during RA, often localized in the skin with pyoderma gangrenosum or in the peripheral nervous system with mononeuritis multiplex, but it can also affect the lungs. Several studies agree that the observed prevalence of rheumatoid vasculitis has progressively reduced over time, reasonably due to earlier diagnosis and better control of the disease [44, 45]. HRCT imaging usually reveals a nodular pattern associated with cavities highlighting antineutrophil cytoplasm antibody- (ANCA-) associated vasculitis as the most likely, and often difficult to rule out, differential diagnosis. Interestingly, a recent case report described lung vasculitis as the first manifestation of RA [46].

#### 2.4.3. Diffuse Alveolar Hemorrhage

Diffuse alveolar hemorrhage (DAH) is defined as intra-alveolar accumulation of red blood cells as a result of severe damage of the alveolar-capillary membrane of the lungs and it is the histological counterpart of the acute respiratory distress syndrome (ARDS). Early stages of DAH are characterized by capillaritis leading to intra-alveolar septa edema and initial extravasation of red blood cells within the septa. Subsequently, the process extends to the alveoli with fibrinoid necrosis, leukocytoclasis, neutrophil recruitment, and intra-alveolar hemorrhage [5]. Despite being a rare condition as a pulmonary manifestation in RA patients, it is worth mentioning DAH for some reasons. First, DAH is a medical emergency and is frequently fatal; secondly, it has been clearly associated with specific conventional synthetic DMARDs (csDMARDs) such as leflunomide [47–49]. The differential diagnosis with ANCA-associated vasculitis is imperative as the latter conditions, mainly microscopic polyangiitis, account for the vast majority of DAH cases observed in clinical practice [50].

## 3. Comorbidities

### 3.1. Amyloidosis

The term amyloidosis includes a wide range of conditions characterized by abnormal extracellular deposition of amyloid in several organs leading to their progressive dysfunction. Amyloid light-chain (AL) amyloidosis is the primary form, while reactive amyloid A (AA) amyloidosis is the isotype that is peculiarly associated with RA and other inflammatory diseases [51]. The prevalence of AA amyloidosis in the general population is below 1% while in RA it ranges from 5 to 20% [52]. The exact pathogenic mechanism of this disease is still unclear, although the most likely hypothesis is that abnormal deposition of AA results from the overproduction of serum AA as a consequence of chronic inflammation [53]. In the vast majority of RA patients suffering from AA amyloidosis, kidneys are the target organ with progressive disfunction culminating in end stage renal disease. Lung involvement is a peculiar feature of AL amyloidosis; however, a consistent number of cases of pulmonary AA amyloidosis have been described [54]. The patterns are the same as lung AL amyloidosis, with diffuse involvement of alveolar septa, pulmonary nodules, or tracheobronchial involvement, the latter being the most commonly symptomatic one, with dyspnea, cough, and hemoptysis. A case of fatal tracheobronchomalacia due to amyloidosis in RA has also been reported [55]. The diagnosis of amyloidosis is confirmed upon demonstration of AA fibrils on biopsy specimens of the target organ with Congo Red staining. Usually, the successful treatment of RA is also helpful to control this comorbidity.

### 3.2. Caplan's Syndrome

Caplan's syndrome, also known as rheumatoid pneumoconiosis, was first described in the 1950s in Wales-based coal miners with RA [56]. It results from the coexistence of mineral coal or silica exposure and RA and is characterized by the presence of rheumatoid and silicotic nodules in the lungs. The prevalence is extremely variable in different geographic areas due to different distributions of minerals, and it changes over time due to the different work conditions [57]. The exact pathogenic mechanism is not entirely clear, and it is established whether pneumoconiosis predisposes to RA or vice versa. Nonetheless, it is evident that most of the patients are seropositive and RF can also be detected in the nodules. According to the severity of the pneumoconiosis, Caplan's syndrome can be defined as Caplan's type (mild pneumoconiosis) or silicotic type (more severe pneumoconiosis, with most nodules being silicotic) [56]. Biopsy specimens reveal that nodules display a central necrotic area surrounded by alternate layers of black coal dust and necrotic tissue. If a layer of inflammatory cells, mainly polymorphonuclear granulocytes and macrophages, is present, the nodule is defined as rheumatoid; otherwise, it is defined as silicotic. At chest X-ray, nodules appear rounded, located at the pulmonary periphery, ranging from 0.5 to 5.0 cm in diameter, with or without small opacities consistent with pneumoconiosis or massive pulmonary fibrosis. In most cases, patients remain asymptomatic and the diagnosis may be incidental upon chest X-ray evaluation for other reasons. Like classical rheumatoid nodules, they can become symptomatic upon complication.

## 4. Drug-Induced Pulmonary Involvement

Although the increase of the therapeutic armamentarium for RA represented a milestone in the management and control of the disease, it also profoundly affected morbidity and mortality with regard to adverse events [58]. In this context, the main concern is related to increased risk of infections. With regard to csDMARDS, methotrexate (MTX) has been associated with a higher risk of infections, but to date the exact burden is not clearly established [58]. Such risk is even higher for bDMARDs compared to csDMARDs as demonstrated by a recent meta-analysis [59]. Furthermore, bDMARDs are associated with a small but significant risk of specific opportunistic infections such as* Pneumocystis jirovecii* pneumonia [60]. Besides the higher risk of infections, several compounds have been pointed out as putatively responsible for exacerbation/worsening of pulmonary manifestations in RA [61]. MTX has been mainly associated with ILD [62–68]. Two recent systematic literature reviews, one of these also with a meta-analysis, demonstrated that MTX is associated with a small but significant increase in total respiratory adverse events and total infectious respiratory events compared with other csDMARDs and bDMARDs [61, 69]. MTX-induced ILD with irreversible fibrotic lesions may develop at any time during treatment and occurs in 48% of patients within 32 weeks of the initiation of MTX [70]. Furthermore, MTX can worsen (namely, increase the dimension) of preexisting rheumatoid nodules [71]. However, the overall number of deaths due to respiratory disease was comparable in MTX-treated and MTX-naïve patients [69]. Conversely, leflunomide (LEF) was not associated with an increased risk of total adverse respiratory events or infectious respiratory adverse events [72]. However, LEF deserves particular attention as long-standing treatment has been associated with the development of DAH [47–49].

With regard to bDMARDs, TNF-*α* blockers are those with more available evidence. Real-life data from the British Society for Rheumatology Biologics Register demonstrated that survival of RA-ILD patients is not affected by the treatment with anti-TNF-*α* agents. However, the proportion of deaths attributable to RA-ILD was higher in patients treated with anti-TNF-*α* therapy. It should be noted that the authors themselves recommend interpreting the latter evidence with caution due to possible methodologic/reporting biases [73]. On the other hand, a study conducted to evaluate the comparison between MTX step-up and anti-TNF-*α* agents in active RA patients failed to observe any difference in incident ILD cases [74]. As far as other bDMARDs are concerned, currently available data are too few to draw any definitive conclusion and, most importantly, to rule out whether a given pulmonary manifestation would occur independently of the treatment [75].

## 5. Conclusion

There are a variety of pulmonary manifestations of RA, including pulmonary parenchymal disease, involvement of the pleurae (pleural thickening and effusions), and inflammation of airways and pulmonary vasculature (vasculitis, PH). The clinical, radiological, and histological spectra of respiratory manifestations in RA reflect chronic immune activation, increased susceptibility to infections (often related to immunomodulatory medications), or direct toxicity from csDMARDs or bDMARDs. At the same time, the prognosis varies depending on the type and severity of this involvement. On this basis, clinicians should remain alert to the possibility of lung disease in all patients with RA, and a multidisciplinary approach is required for correct management of these patients. Many aspects in the pathogenesis of some manifestations remain unclear and their understanding in the near future would allow developing novel effective and selective therapeutic strategies.
